# Supplementary material for: Natural Antibacterial Compounds with Potential for Incorporation into Dental Adhesives: A Systematic Review
Source: Polymers (Basel). 2024 Nov 20;16(22):3217. doi: 10.3390/polym16223217 (PMC11598765; doi:10.3390/polym16223217)
Supplement: Supplementary file 1 [file polymers-16-03217-s001.zip › Table S1.pdf]

SUPPLEMENTARY MATERIALS

Table S1. RoB analysis of the studies using the RoBDEMAT tool.

Author	D1: Bias in Planning and Allocation			D2: Bias in Sample/Specimen Preparation		D3: Bias in Outcome Assessment		D4: Bias in Data Treatment and Outcome Reporting	
	**Control Group**	**Randomized Samples**	**Sample Size Calculation**	**Standardized Samples and Materials**	**Identical Experimental Conditions**	**Standardized Testing Procedures**	**Operator blinded**	**Statistical Analysis**	**Reporting Outcomes**
Du et al. (2012)	Reported	Not applicable	Not reported	Insufficiently reported	Reported	Insufficiently reported	Not reported	Reported	Reported
Elsaka (2012)	Reported	Not applicable	Not reported	Reported	Reported	Reported	Not reported	Reported	Reported
Peralta et al. (2013)	Reported	Insufficiently reported	Not reported	Reported	Reported	Reported	Not reported	Reported	Reported
Geraldeli et al. (2017)	Reported	Not applicable	Not reported	Insufficiently reported	Reported	Insufficiently reported	Not reported	Reported	Insufficiently reported
Yang et al. (2017)	Reported	Not applicable	Not reported	Insufficiently reported	Reported	Reported	Not reported	Reported	Reported
Su et al. (2018)	Reported	Not applicable	Not reported	Insufficiently reported	Reported	Reported	Not reported	Reported	Reported
Rezaeian et al. (2019)	Reported	Not applicable	Not reported	Insufficiently reported	Reported	Insufficiently reported	Not reported	Reported	Insufficiently reported
Dias et al. (2020)	Reported	Not applicable	Not reported	Reported	Reported	Reported	Not reported	Reported	Reported
Leyva del Rio et al. (2020)	Reported	Not applicable	Not reported	Reported	Insufficiently reported	Reported	Not reported	Reported	Reported
Zhao et al. (2020)	Reported	Not applicable	Not reported	Insufficiently reported	Reported	Reported	Not reported	Reported	Reported
Ribeiro et al. (2021)	Reported	Insufficiently reported	Not reported	Insufficiently reported	Reported	Reported	Not reported	Reported	Reported
de Oliveira Souza et al. (2022)	Reported	Not applicable	Not reported	Reported	Reported	Reported	Not reported	Reported	Insufficiently reported
Yao et al. (2022)	Reported	Not applicable	Not reported	Reported	Reported	Reported	Not reported	Reported	Reported
